# Supplementary material for: Highly Sensitive Temperature Sensors Resulting from the Luminescent Behavior of Sm3+-Doped Ba2MgMoO6 High-Symmetry Double-Perovskite Molybdate Phosphors
Source: Materials (Basel). 2024 Apr 19;17(8):1897. doi: 10.3390/ma17081897 (PMC11052038; doi:10.3390/ma17081897)
Supplement: Supplementary file 1 [file materials-17-01897-s001.zip › materials-2960975-supplementary.pdf]

Supplementary

Highly sensitive temperature sensors resulting from the luminescent behavior of Sm^{3+}-doped Ba_2MgMoO_6 high symmetry double-perovskite molybdate phosphors

N. Miniajluk-Gaweł[a*], B. Bondzior[a], M. Ptak[a], P. J. Dereń[a*]

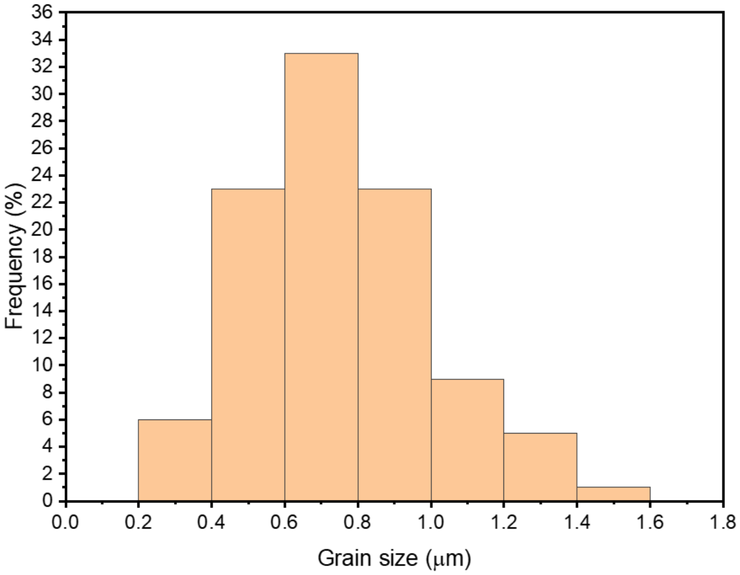

Figure S1. Histogram of the average crystal size.

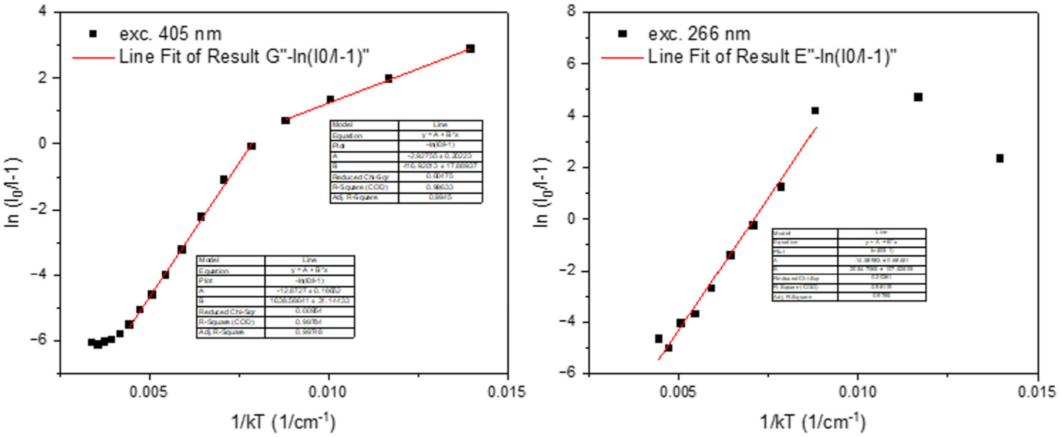

Figure S2. Fitting of an Arrhenius equation to temperature-dependent emission intensity of BMM:Sm^{3+} excited at 405 nm (left) and 266 nm (right).
